# Experimental Investigation on Microstructure Alteration and Surface Morphology While Grinding 20Cr2Ni4A Gears with Different Grinding Allowance Allocation

**DOI:** 10.3390/ma16186111

**Published:** 2023-09-07

**Authors:** Rong Wang, Size Peng, Bowen Zhou, Xiaoyang Jiang, Maojun Li, Pan Gong

**Affiliations:** 1 Hunan Xingtu Aerospace and Spacecraft Manufacturing Co., Ltd., Zhuzhou 412000, China; 2 State Key Laboratory of Advanced Design and Manufacture for Vehicle Body, Hunan University, Changsha 410082, China; 3 State Key Laboratory of Materials Processing and Die & Mold Technology, School of Materials Science and Engineering, Huazhong University of Science and Technology, Wuhan 430074, China

**Keywords:** allowance allocation, microstructure, surface morphology, form grinding

## Abstract

Transmission gear is a key component of vehicles and its surface integrity affects the safety of the transmission system as well as the entire mechanical system. The design and optimization of allowances in form grinding are important for improving dimensional accuracy and machining efficiency during the manufacturing of heavy-duty gears. This work aims to investigate the effects of grinding allowance allocation on surface morphology, grinding temperature, microstructure, surface roughness, and microhardness fluctuation during the form grinding of 20Cr2Ni4A gears. Results indicated that grinding temperature was primarily influenced by rough grinding involving significant grinding depths exceeding 0.02 mm. The ground surface exhibited slight work hardening, while thermal softening led to a reduction in microhardness of around 40 HV. Ground surface roughness Ra varied from 0.930 μm to 1.636 μm, with an allowance allocation of the last two passes exerting the most significant influence. Analysis of surface and subsurface microstructures indicated that a removal thickness of 0.02 mm during fine grinding was insufficient to eliminate the roughness obtained from rough grinding. Evident ridges, gullies, and surface defects such as material extraction, adhesion, and plastic deformation were also observed. The proposed grinding strategy was validated in practical manufacturing with good surface quality and geometrical accuracy.

## 1. Introduction

Heavy-duty gear components are extensively utilized in vehicles, and the demand for efficient and precise machining of these ones is continuously growing alongside industrial advancements. Form grinding plays a pivotal role in the manufacturing and processing of gears. Prior to the grinding process, samples are processed with forging, rough machining, and heat treatments. Consequently, grinding is the final process to ensure the ultimate accuracy and surface quality of gear products. To improve manufacturing efficiency, the total grinding allowance is designed to be minimized due to the lower material removal rate and the higher specific energy associated with grinding compared to other machining methods such as turning and milling [1]. Both the grinding forces and residual stress from previous heat treatments will affect machining quality and geometrical accuracy. Previous studies have shown that allowance allocation in grinding is highly related with stress generation, highlighting the importance of considering allowance allocation in different grinding stages to enhance part accuracy [2].

Research works investigated approaches to improve machining accuracy by optimizing machining allowances. For instance, Batueva et al. [3] proposed a machining model stabilizing the cutting force component by adjusting the machining allowance of complex areas with varying shapes on the machined surface, thereby enhancing machining accuracy. Ruzzi et al. [4] analyzed the effects of grinding parameters on surface integrity during the surface grinding of Inconel alloys, where the design of the experiment method was applied using grinding speed, work speed, grinding depth, and up/down grinding as variations. Results showed that grinding speed was the most significant factor affecting work hardening. Luu et al. [5] devised a novel method for designing a conical skiving cutter that allowed pre-defined grinding allowance for skived gears by modifying the normal rack, resulting in a uniform grinding allowance on active involute sections. Lv et al. [6] presented a novel path route planning method that generated the grinding route based on residual height error compensation, a proposed geometric algorithm, and the machining allowance threshold. Optimizing the machining allowances can enhance efficiency and reduce energy consumption, thereby supporting sustainable development in the manufacturing industry [7]. Guo et al. [1] introduced a systematic energy-efficient method based on minimizing energy consumption while ensuring acceptable surface roughness to quantify grinding stock allowances in the turning–grinding process, which was validated by reducing energy consumption up to 16.6%. Hood et al. [8] used electroplated diamond superabrasive wheels for grinding an intermetallic alloy in order to improve both the grinding efficiency and surface quality. Guerrini et al. [9] conducted a gear machining experiment by varying the skiving–grinding allowance allocation ratios and optimizing the process parameters, demonstrating the feasibility of dry grinding for gear machining while ensuring accuracy and avoiding grinding burn. Li et al. [10] aimed to achieve high-efficiency and high-quality glass–ceramic grinding by optimizing the appropriate grinding parameters based on the study of parameter influence on processing behavior during rough grinding, semi-fine grinding, and fine grinding processes.

However, the research on machining allowances have not comprehensively addressed the dual goals of improving machining efficiency and accuracy. For heavy-duty gears, the integrity of ground surface directly affects contact, wear, and the generation of cracks, ultimately influencing the fatigue life of the whole component [11]. Guerrini et al. [9,12] proposed a defined threshold energy level to prevent grinding burns on gear surfaces via a dry grinding experiment, analyzing the effects of different processing parameters on the white layer and hardness gradient in the subsurface. Riebel et al. [13] studied the influence of wheel groove depth/width on the grinding performance during creep grinding and found that coolant-induced force was significant for improving the grinding performance. Murtagian et al. [14] proposed a grinding model to analyze the effects of abrasive particle size and shape, grinding feed, and the depth of the cut on subsurface plastic deformation depth. Yang et al. [15] investigated the effect of grinding parameters on surface roughness and elucidated the formation mechanism of plastic deformation and defects on ground surface. Tao et al. [16] proposed a transient analog model to simulate the transient material removal behavior in continuous forming grinding, predicting the grinding morphology of the tooth surface. They found that the top tooth surface was relatively smoother than the root one, and the surface roughness decreased along the feed direction.

Based on the aforementioned literature review, prior research focusing on ground surface integrity have not adequately examined the influence of grinding allowance allocation on surface quality. To fill this gap, the effects of grinding allowance allocation on grinding temperature, surface roughness, and microhardness fluctuation were analyzed. The research work aimed to study the influence characteristics/mechanisms of different grinding strategies on surface morphology and microstructure and to further understand the corresponding material removal mechanisms during the form grinding process. Several principles for grinding allowance allocation in the process are summarized, providing guidance for optimizing the manufacturing process of gear products and improving production efficiency.

## 2. Experimental Works

### 2.1. Workpiece Material and Equipment

A raw vehicle transmission gear made of 20Cr2Ni4A was selected for the experiment, and it was cut into 12 small pieces via the wire electrical discharge machining technique, which generated very limited heat and defects for the grinding samples and would not further affect the grinding performance. The chemical composition of 20Cr2Ni4A is presented in Table 1. The material exhibits good toughness due to its low carbon content. The gear has undergone carburizing and quenching processes, resulting in the formation of a carburized layer on the surface. This carburized layer enhances the material’s abrasion resistance and imparts high hardness. Microhardness measurements indicated that the maximum hardness of the surface layer for the gear sample used in this study was ~650 HV. The effective thickness of the carburized layer, characterized by the microhardness higher than 550 HV, was 1100 μm. 

**Figure 1:**
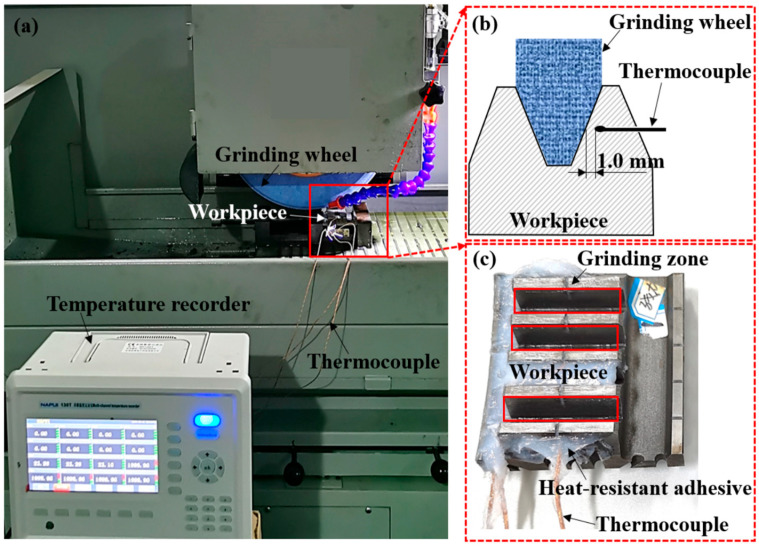
(**a**) Grinding equipment, (**b**) schematic diagram of temperature measurement using thermocouple and (**c**) workpiece sample for grinding trials.

The gear grinding trials were performed using a three-axis automatic hydraulic high-precision grinding machine (DY-510ASM, China). The machine had a maximum rotation speed of 4000 r/min and a rated power of 7.5 kW. The gear tooth profile was obtained using an alumina forming grinding wheel with #80 abrasive mesh. The maximum diameter of the grinding wheel was 350 mm, and its profile coincided with the gear involute. Before each trial, the grinding wheel was dressed to ensure consistent working conditions throughout the grinding process. During the grinding process, temperature signals were captured using a GG-K-30-1000-CZ type thermocouple and a multiple-channel temperature recorder (NAPUI-HE130T-16, China), which has the acquisition frequency of 0–10,000 Hz and the resolution of 0.01 °C. As the grinding time in each pass was very short, the acquisition frequency was set at 8000 Hz in order to obtain real-time temperature variations. To position the thermocouple, a narrow groove with a width of 1.0 mm was cut in the middle of the tooth surface. The thermocouple with a diameter of 1.0 mm was placed in the groove and sealed/fixed using heat-resistant waterproof adhesive, as shown in Figure 1. The sensor end of the thermocouple was positioned ~0.5 mm away from the tooth surface. Temperature measurements were recorded at a sampling frequency of 1 Hz. A white light interference was employed to observe ground surface morphology. A roughness meter was used to measure surface roughness (Ra), with each sample being measured three times, followed by taking the average value. The teeth of each grinding set were cut into samples and embedded in resin. The cross-section of the ground surface was ground and polished. Subsequently, it was corroded using a 4% nitric acid–alcohol solution for 15 s. To analyze the material microstructure, a scanning electron microscope (COXEM EM-30N, China) was utilized. The microscope was operated with magnifications of ~2000–5000 times. Additionally, a hardness tester was used to measure the microhardness, with 1000 g load for 10 s.

### 2.2. Grinding Parameters

**Figure 2:**
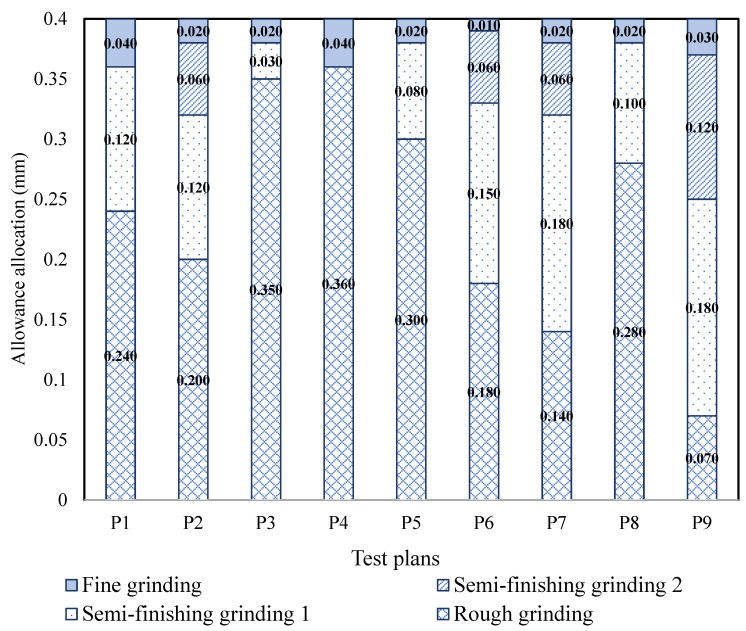
Allowance allocation in different grinding stages.

In the gear manufacturing process, it is crucial to eliminate any deformations caused by heat treatment and ensure the dimensional accuracy of the final product. In this study, the total material removed from the raw sample to the finished one was set as 0.400 mm. The grinding parameters were selected according to on-site machining parameters. The grinding parameters commonly used on the grinding machine range from 0.19 m/s to 0.35 m/s for the feed speed, 0.01 mm to 0.04 mm for the grinding depth, and the spindle speed is 900 r/min to 4000 r/min. Three different combinations of allowances were selected to design the grinding allowance allocation strategy. These included rough grinding–semi-fine grinding–fine grinding (referred to as P1, P3, P5, and P8), rough grinding–semi-fine grinding 1—semi-fine grinding 2—fine grinding (referred to as P2, P6, P7, and P9), and rough grinding–fine grinding (referred to as P4), as shown in Figure 2. Nine grinding allowance allocation plans were designed based on these combinations, with details listed in Table 2. To investigate the influence of improving machining efficiency in the rough grinding and semi-fine grinding stages on grinding temperature and surface integrity, the grinding depth for the rough grinding stage was set at 0.025 mm, 0.03 mm, and 0.035 mm, respectively. The grinding depth for each stage decreased relating to the previous stage. 

## 3. Results and Discussion

### 3.1. Grinding Temperature

**Figure 3:**
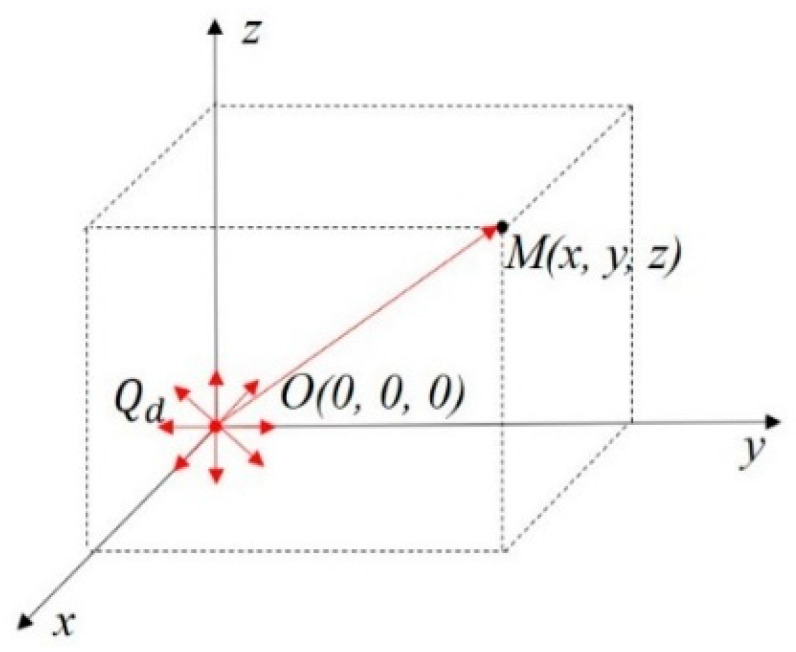
Schematic diagram of instantaneous point heat source temperature field.

According to the recorded grinding temperature and the details of allowance allocation for different grinding trials, it was found that the grinding depth and the number of consecutive grinding times in different grinding stages showed a significant influence on grinding temperature. Due to the negative rake angle of the abrasive particles, a large quantity of energy was generated in the contact area between the grinding wheel and the workpiece during grinding with a large cutting depth, and most of the energy was converted to a high temperature in the grinding zone [17]. Additionally, continuous grinding on the tooth grooves multiple times blocked the fluid entering the contact area and promoted grinding temperature [18]. According to the grinding temperature model established by Yang et al. [19], when a transient point heat source $Q_{d}$ occurred at O(0,0,0) in an infinite homogeneous space, as shown in Figure 3, the temperature change at any point M(x,y,z) in the space over time τ could be calculated as:

(1) $$T=\frac{Q_{d}}{c\rho {\left(4\pi \alpha \tau \right)}^{3/2}}e^{-\frac{x^{2}+y^{2}+z^{2}}{4\alpha \tau }}$$

In which T was the temperature change, c was the specific heat capacity of workpiece material, ρ was the density of workpiece material, and α was the thermal conductivity.

**Figure 4:**
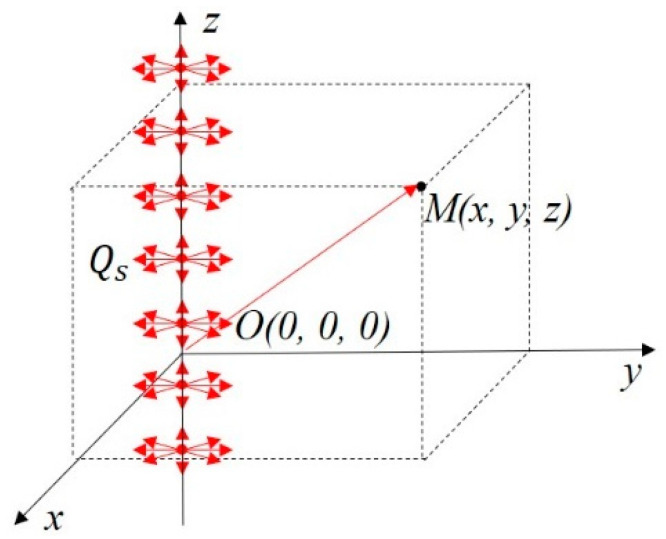
Schematic diagram of temperature field of instantaneous infinite linear heat source.

As shown in Figure 4, when the heat source was replaced by an instantaneous infinitely long linear heat source along the *z*-axis, and the uniform heat output was $Q_{s}$, the temperature change to any point M(x,y,z) in space over time τ was the integral of the point heat source along an infinitely long straight line, as shown below:

(2) $$T=\frac{Q_{s}}{c\rho {\left(4\pi \alpha \tau \right)}^{3/2}}e^{-\frac{x^{2}+y^{2}}{4\alpha \tau }}\int_{-\infty }^{+\infty }e^{-\frac{{\left(z-z_{i}\right)}^{2}}{4\alpha \tau }}dz_{i}$$

Equation (2) can be simplified as

(3) $$T=\frac{Q_{s}}{c\rho (4\pi \alpha \tau )}e^{-\frac{x^{2}+y^{2}}{4\alpha \tau }}$$

**Figure 5:**
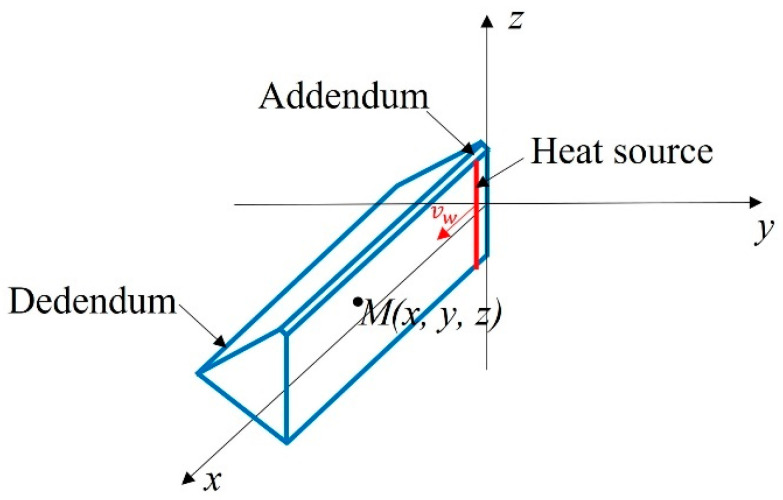
Schematic diagram of simplified tooth surface moving line heat source.

From Equation (3), it can be seen that the temperature rise at any point was only related to the distance from that point to the linear heat source. In order to facilitate calculation, the gear involute in this experiment was simplified to a straight line. Since the width of the tooth surface was much smaller than the diameter of the grinding wheel, the linear speed of each part of the grinding wheel was considered to be the same. Thus, as shown in Figure 5, the grinding area was simplified as a constant heating linear segment where the heat source moves uniformly on the tooth surface with a heating power of $Q_{s}$.

According to Equation (3), in a flash of $d\tau_{i}$ the temperature change dT caused by the heat source differential section $q_{s}d\tau_{i}$ at point M was

(4) $$dT=\frac{q_{s}d\tau_{i}}{c\rho (4\pi \alpha \tau )}e^{-\frac{{\left(x-v_{w}\tau_{i}\right)}^{2}+y^{2}}{4\alpha \tau }}$$

**Figure 6:**
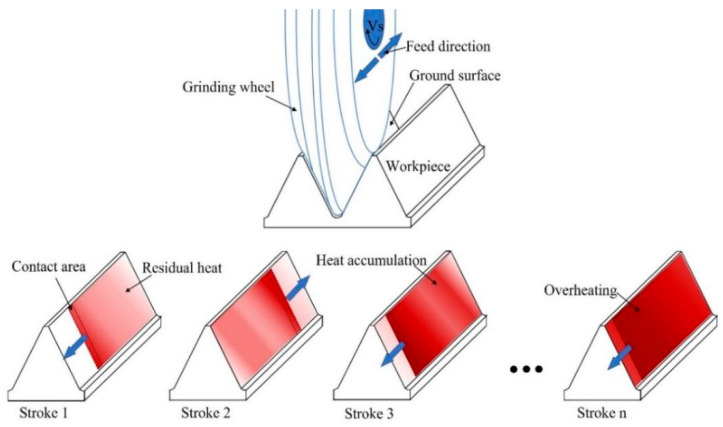
Schematic diagram of heat accumulation mechanism in continuous grinding.

The heat source started moving from the moment $\tau_{i}$ and continued moving for a period τ to reach another moment t. In the model of Figure 6, the distance from the heat source to the point M(x,y,z) on the x-axis was defined as $X=x-v_{w}t$, where $v_{w}$ was the feed speed. Equation (4) could be transformed to Equation (5):

(5) $$dT=\frac{q_{s}d\tau_{i}}{c\rho (4\pi \alpha \tau )}e^{-\frac{{\left(X+v_{w}\tau \right)}^{2}+y^{2}}{4\alpha \tau }}$$

According to Equation (5), during the movement of the heat source from the moment to move to the moment t, the temperature rise of M(x,y,z) caused by the moving constant linear heat source could be calculated as:

(6) $$T=\frac{q_{s}}{4\pi \lambda }\int_{0}^{t}\frac{1}{\tau }e^{-\frac{{\left(X+v_{w}\tau \right)}^{2}+y^{2}}{4\alpha \tau }}d\tau_{i}$$

Equation (6) indicates that the temperature at the tooth surface and any internal point is continuously influenced by the heat source throughout the grinding process, from the beginning of a grinding stroke to the end of it. Therefore, even after the grinding area has passed through a measurement point, the heat source still affects the temperature at that point. Furthermore, due to the inability to dissipate heat instantaneously, the grinding heat conducted into the workpiece persists for a certain period of time after grinding. The contact area between the grinding wheel and the workpiece is considered a continuous heat source, resulting from the heat generated by friction and cutting. As depicted in Figure 6, during a grinding stroke, the heat source moves from one end of the tooth to the other, with most of the heat being transferred to the workpiece, leading to an increase in grinding temperature. When a grinding stroke is completed and immediately followed by the next one, a new heat source is generated and moves uniformly across the tooth surface. In this work, the workbench’s movement track length is 200 mm, and the feed speed is 0.19 m/s, resulting in an interval of approximately one second between two consecutive grinding strokes. As a result, at the start of a new grinding stroke, the temperature at the previously ground surface has not yet decreased to the ambient temperature, and the residual heat in the workpiece has not dissipated completely. Consequently, in the new stroke, more heat is generated and transferred into the workpiece, accumulating with the residual heat. With continuous grinding, the accumulated heat in the workpiece leads to high grinding temperatures. This phenomenon is a result of the heat accumulation and insufficient dissipation between consecutive grinding strokes, contributing to the generation of elevated grinding temperatures.

**Figure 7:**
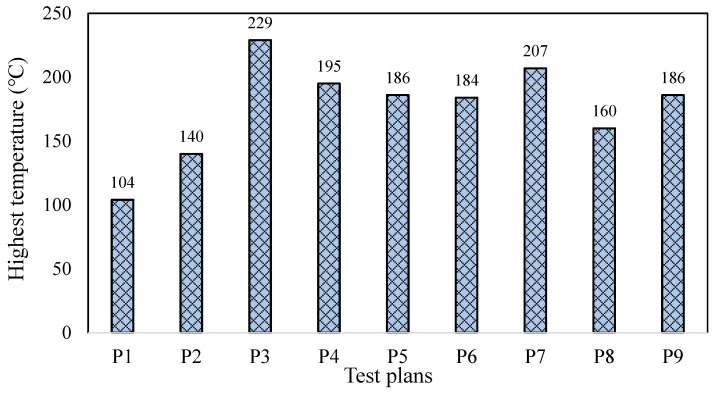
Maximum temperature recorded in all trials with different test plan/allowance.

Figure 7 presents the maximum temperatures recorded during all grinding trials. As the temperature changes during grinding passes were relatively low and the grinding time in each pass was very short, the temperature variation/duration over time was not presented in this work. The maximum temperatures ranged from 104 °C to 229 °C, indicating that the different grinding allowance allocation strategy had a significant impact on it. Specifically, trial P3 exhibited a considerably higher temperature compared to the other ones. The allowance in P3 was divided into three stages, with a grinding depth of 0.025 mm in the rough grinding stage (14 passes), 0.015 mm in the semi-fine grinding stage (2 passes), and 0.01 mm in the fine grinding stage (2 passes). As the grinding depth increased, more abrasive particles were involved in the cutting process, generating and transferring more heat into the workpiece [20,21,22]. Additionally, the limited thermal conductivity of the workpiece material and the limited time interval between each grinding stroke led to heat accumulation. The unique characteristic of the allowance allocation strategy in P3 was the removal of a substantial amount of material via continuous grinding with a large grinding depth in the rough grinding stage, accounting for approximately 90% of the total allowance, which resulted in a sharp increase in temperature due to the significant accumulation of heat. For trial P7, the allowance was divided into four stages including 0.035 mm in the rough grinding stage (4 passes), 0.03 mm in semi-fine grinding stage 1 (6 passes), 0.015 mm in semi-fine grinding stage 2 (4 passes), and 0.01 mm in the fine grinding stage (2 passes). Similar to the strategy in trial P3, a significant amount of material was removed via continuous grinding with a large grinding depth in the rough grinding and semi-fine grinding stage 1, with a total of 10 grinding strokes having a grinding depth of not less than 0.03 mm, accounting for 80% of the total allowance, which subsequently produced a relatively high grinding temperature.

Trial P1 exhibited the lowest temperature among all trials, as the allowance allocation ratio for each stage was rough grinding: semi-fine grinding 1: fine grinding = 6:3:1, and the grinding depth for each stage did not exceed 0.02 mm. The grinding depth in the rough grinding stage was only 0.02 mm, with 12 grinding strokes. Compared to trial P3, the generation and accumulation of grinding heat were significantly reduced, leading to a lower grinding temperature. Trial P2 showed a slightly higher temperature when the allowance allocation ratio for each stage was rough grinding: semi-fine grinding 1: semi-fine grinding 2: fine grinding = 10:6:3:1. Except for rough grinding, the grinding depth for each stage did not exceed 0.02 mm. In the rough grinding stage, the grinding depth was 0.025 mm, with 8 grinding strokes resulting in a maximum temperature of 140 °C.

### 3.2. Ground Surface Topography

**Figure 8:**
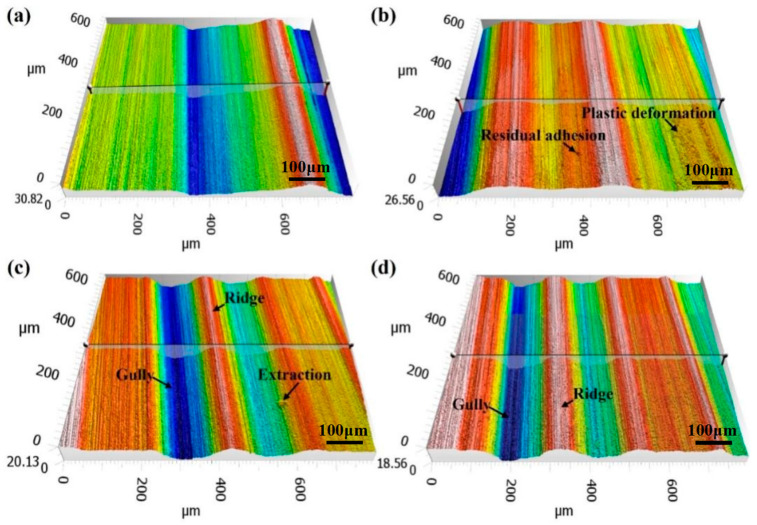
Typical ground surface topography recorded from samples in trials (**a**) P2, (**b**) P3, (**c**) P5, and (**d**) P7.

The morphology of the ground gear surface plays a crucial role in determining the surface quality, contact fatigue strength, crack resistance, and transmission stability of the final product. The precision grinding parameters used in the final grinding pass have a significant impact on surface integrity and gear performance. Figure 8 presents the three-dimensional morphology of the ground surfaces obtained from four trials with different grinding allowance allocation. Although no macroscopic cracks were observed in the profiles, it is difficult to determine whether the microcracks were presented due to limited magnification. Similarly, it is challenging to assess grinding burns based solely on the color or burning traces on the surface. Serious grinding burns are typically caused by high temperatures, resulting in residual tensile stresses within the surface layer, which can lead to the formation of microcracks [13]. The ground surface profiles mainly consist of flat surfaces, gullies, and ridges. The formation of gullies and ridges can be attributed to material plastic deformation induced by grinding heat, where the material is pushed to both sides by abrasive particles. Another possible reason for the formation of gullies and ridges is the presence of surface fluctuations caused by a large grinding depth in the preceding passes, which cannot be fully eliminated in the final finish grinding stage. For instance, in the case of trial P5, a substantial amount of material (0.3 mm) was removed in the rough grinding stage over 10 passes. In the subsequent semi-fine grinding stage, 0.08 mm of material was removed over four passes, and only two passes were used for surface repair in the fine grinding stage, with a total grinding depth of 0.01 mm. It is evident that the surface fluctuations cannot be completely mitigated, resulting in visible gullies and ridges on the ground surface of the samples from trial P5.

Various defects can be observed on the ground surface, including surface residual adhesion and plastic deformation, as shown in Figure 8b, as well as the material extraction depicted in Figure 8c. The extraction of granular material and the presence of residue adhesion are likely the result of material adhesion to the grinding wheel due to the influence of grinding heat. These defects and surface plastic deformation are closely related to high temperatures. Overall, the surface morphology and defects observed on the ground surface provide valuable insights into the quality of the grinding process and the potential influence of parameters on the resulting surface characteristics.

**Figure 9:**
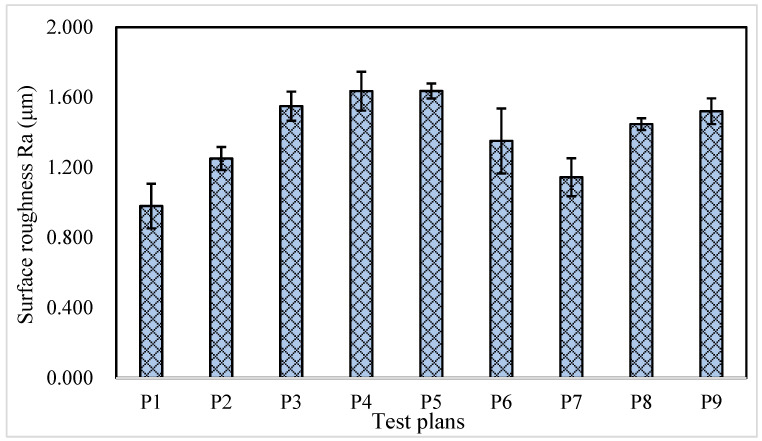
Surface roughness Ra for all trials with different grinding allowance allocation/test plans.

The surface roughness (Ra) values of all trials with different grinding allowance allocations are presented in Figure 9, with the lowest one recorded in trial P1. It was observed that from rough grinding to fine grinding, the allowance allocated for each pass and the grinding depth of each stage gradually decreased in a gradient manner in trial P1. This allocation feature for each grinding pass contributed to improving the surface quality achieved in the preceding pass. Comparing the allocation characteristics in trials P5 and P7, it can be observed that the allocation in the last two passes largely determined the final surface roughness. It is evident that the surface roughness obtained by P7 (0.015 mm × 4 grinding strokes + 0.010 mm × 2 grinding strokes) is smaller than that of P5 (0.020 mm × 4 grinding strokes + 0.010 mm × 2 grinding strokes). Furthermore, comparing the Ra value of trials P6 and P7 reveals that at a grinding depth of 0.005 mm in the finish grinding stage, the rough surface resulting from the previous grinding pass cannot be completely polished in just two grinding strokes without increasing the number of ones. The low roughness value in trial P1 indicates that a smooth surface can be achieved with a grinding depth of 0.01 mm in the fine grinding stage. The roughness values recorded in trials P2 and P7 suggest that adopting a grinding depth of 0.015 mm in the final semi-finish grinding stage is feasible. This implies that the ground surface roughness can be improved without extending the machining time. On the premise that fine grinding is capable of repairing the surface quality obtained from previous grinding passes, processing time can be saved in the rough grinding stage. This approach is similar to the method reported by Li et al. [23], where fine grinding was employed to remove the burn layer caused by rough grinding and enhance the overall grinding efficiency.

### 3.3. Microstructure Morphology

**Figure 10:**
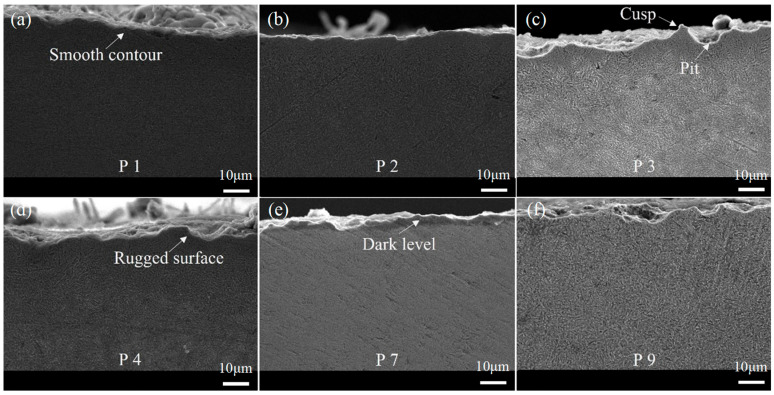
Microstructure morphology of subsurface from samples with different grinding allowance allocations in trials (**a**) P1, (**b**) P2, (**c**) P3, (**d**) P4, (**e**) P7, and (**f**) P9.

Typical microstructure morphology within the subsurface layer is shown in Figure 10. The profiles of ground subsurface in trials P1 and P2 exhibit relatively smooth contours with no obvious pits or bumps. This can be attributed to the polishing effect brought about by the last two grinding passes, which improved the surface roughness. In contrast, the surface profiles in trials P3 and P4 are generally rough, and they consist mainly of short smooth lines, bumps, and pits. In the case of the samples from trial P3, significant sharp cusps and pits are observed in the subsurface, primarily resulting from plastic deformation at high temperature. A darker-colored thermally softened layer is visible below the contour, consistent with the measurement of the high grinding temperature. This indicates that a large amount of grinding heat penetrated into the interior of the workpiece during the rough grinding stage, affecting the deeper base material. The contour of the sample from trial P7 is smoother compared to the one in trial P3, mainly due to the polishing effect in the second semi-finishing and finishing stages. However, a dark layer caused by thermal effects can still be observed, suggesting that a significant amount of grinding heat infiltrated the workpiece and affected the deep materials during the rough grinding stage. In the case of trial P9, the sample contour is distributed with numerous small bumps and pits. These are primarily caused by the large grinding depth in the rough grinding stage. In the fine grinding stage, the polishing effect on the rough ground surface is not sufficient with a grinding depth of 0.015 mm. Defects such as pits, cusps, dark layers, and plastic deformation are present, which can be attributed to the thermal effects and polishing effect determined via the allowance allocation. Overall, the subsurface profiles provide visual evidence of the effects of allowance allocation on surface quality and the subsurface characteristics of ground samples. Smooth contours and minimal defects are observed in trials with appropriate allowance allocation, while rough surfaces and various defects are apparent in those with inadequate allowance allocation or excessive grinding depths.

**Figure 11:**
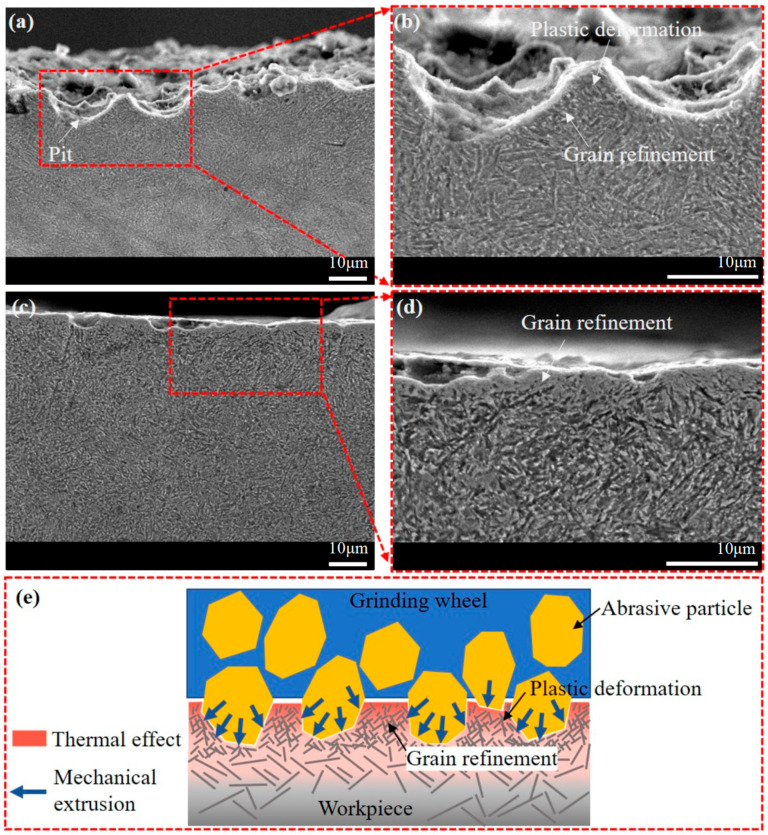
Subsurface morphology of samples from trials (**a**,**b**) P3 and (**c**,**d**) P9, and (**e**) schematic diagram showing mechanism of plastic deformation and grain refinement.

Figure 11 presents a detailed morphology of the ground subsurface from trials P3 and P9 with higher magnifications. In Figure 11a,b, sharp tooth-shaped plastic deformation is evident in the surface profile, contributing to surface irregularities. The grains beneath the ground surface are relatively fine, which may be the result of grain refinement caused by the extrusion of the grinding wheel. In Figure 11d, a layer with a thickness of several microns can be observed below the ground surface, and the grain morphology within this layer is unclear. This layer is typically known as the grinding white layer, which results from dislocation and grain refinement caused by grinding heat and mechanical extrusion. The white layer can improve surface hardness, but it can also increase brittleness, and a thermally softened layer tends to form beneath it. Although phase transformation can also contribute to the generation of this special layer, the measurement results of grinding temperature suggest that it is challenging for the general grinding process to reach temperatures necessary for phase transformation. Figure 11e illustrates the mechanism of plastic deformation and grain refinement. During abrasive cutting and plowing, materials in front of the abrasive’s movement are compressed, forming chips that flow to both sides. It generates a significant amount of grinding heat, which further intensifies plastic deformation. With the occurrence of dislocations and slippage, the original crystal stacking structure of the workpiece material is disrupted, causing large grains to break into smaller ones and form new stacking structures. Grain refinement occurs within a limited depth below the surface due to the small grinding depth in the finish grinding stage. Grain refinement reduces the volume of grains, thereby improving surface microhardness and strength. However, compared to the mechanical effects, the depth of grinding heat conduction is relatively deeper, which may lead to the formation of a heat-softened layer with coarse grains beneath the hardened layer.

**Figure 12:**
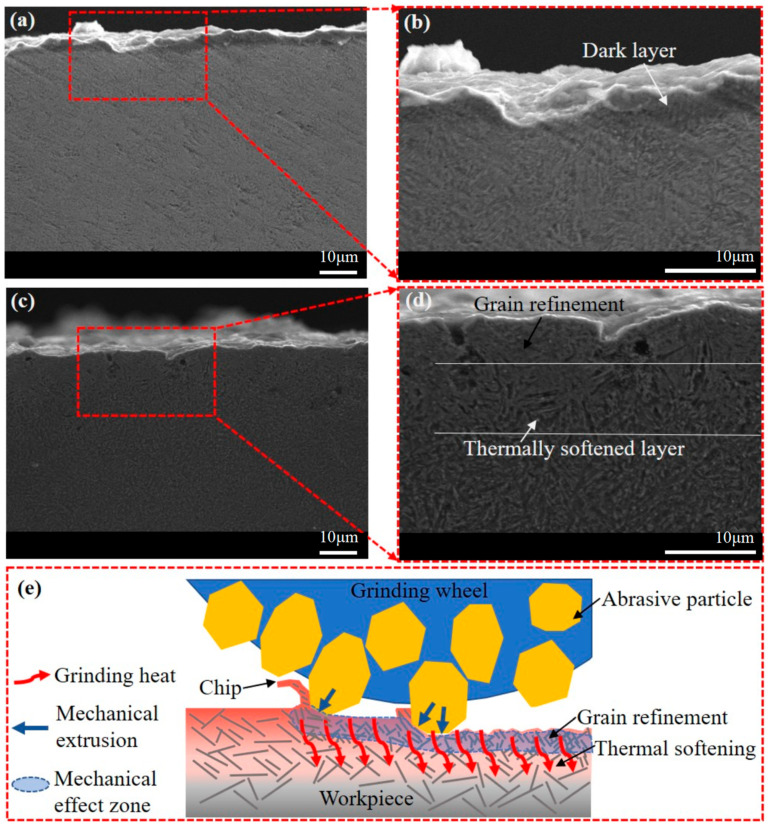
Subsurface morphology of samples from trials (**a**,**b**) P7 and (**c**,**d**) P4, and (**e**) schematic diagram showing generation mechanism of thermal effect.

As shown in Figure 12, the dark layer observed on the ground surface from trial P7 indicates the presence of thermal effects. The high grinding temperature resulting from a grinding depth of 0.035 mm in the rough grinding stage may have caused grinding burn. Although the burn layer was partially removed during the second semi-finishing grinding and fine grinding stages, a shallow dark layer remained. Figure 12c,d illustrate the subsurface morphology of the samples from trial P4. The shallowest layer represents a grain refining layer generated via mechanical extrusion, followed by a thermally softened layer with coarse grains. Deeper into the subsurface, the grains become finer. This change suggests that the subsurface from trial P4 can be divided into three layers including a mechanically affected layer, a thermally affected layer, and an unaffected layer. Figure 12e depicts the mechanism relating to this effect, indicating that thermal effects occur in a deeper layer compared to the mechanical ones. This finding supports the understanding that the depth of grinding heat conduction is probably greater than the depth of mechanical extrusion and grain refinement.

**Figure 13:**
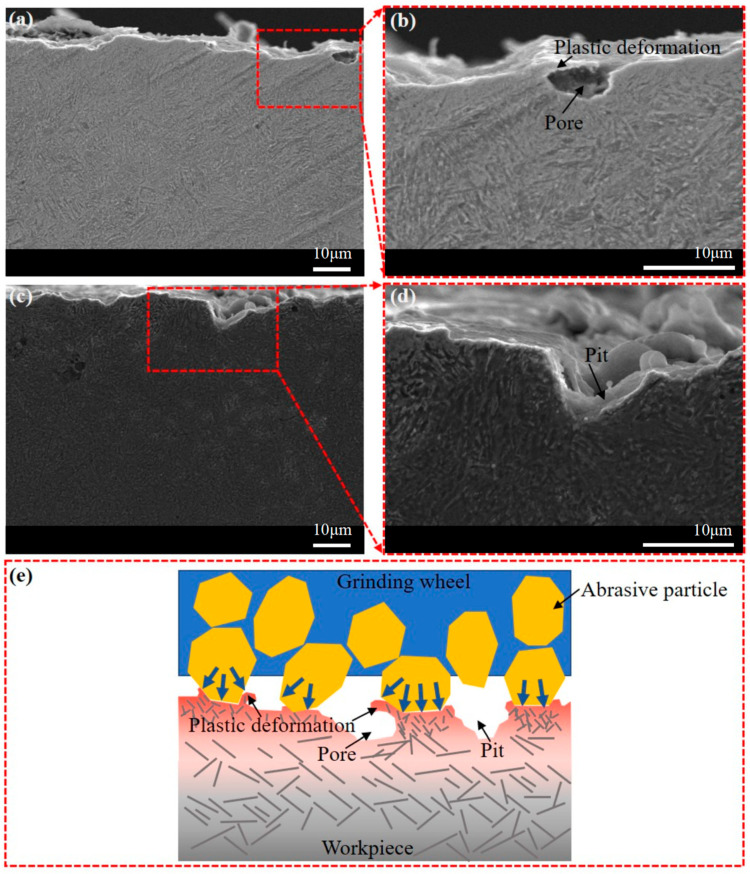
Subsurface morphology of samples from trials (**a**,**b**) P8 and (**c**,**d**) P5, and (**e**) schematic diagram showing generation mechanism of pores and pits.

Figure 13 depicts the presence of pores and pits observed on the subsurface and the corresponding mechanisms. Pits on the grinding surface can be attributed to abrasive particles embedding into the material, as indicated by previous research [15]. Additionally, thermal stress caused by grinding heat can accelerate the wear of the grinding wheel. Another possible reason for the presence of pores and pits is the transformation of deep gaps and ridges formed during the rough grinding stage into holes and pits via mechanical extrusion during the fine grinding stage. For trials P5 and P8, they had a total grinding allowance of 0.02 mm accounting for only 5% of the total material thickness, where it becomes challenging to achieve the desired surface quality if the damaged surfaces cannot be effectively repaired during the fine grinding stage. Figure 13e illustrates a possible mechanism for the generation of pores and pits. In the fine grinding stage, with a small grinding depth, the proportion of scratching and plowing increases. The material in the ridges flows to both sides under the pressure of the abrasive particles but is not completely removed. Meanwhile, the grooves adjacent to the ridges are not sufficiently squeezed. As a result of plastic deformation, gullies remain on the ground surface, and the cross-section surface appears as pits and pores.

### 3.4. Microhardness Variations

**Figure 14:**
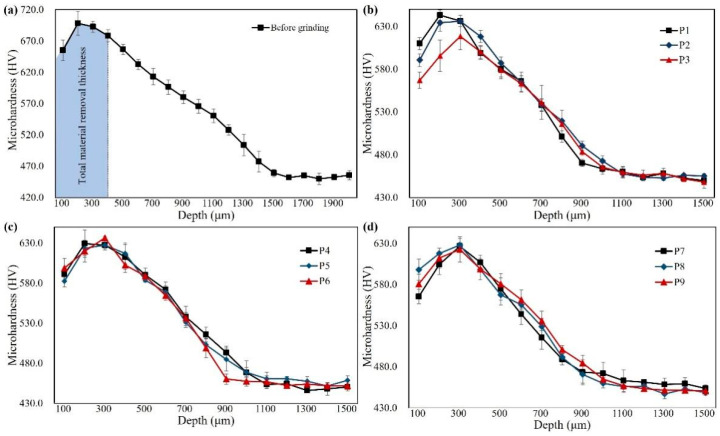
Microhardness variation within the subsurface layer when grinding quenched gear samples with different grinding allowance allocation, including samples (**a**) before grinding and from trial (**b**) P1 to P3, (**c**) P4 to P6, and (**d**) P7 to P9.

Figure 14 presents the microhardness gradient curve of the ground subsurface from all trials with different grinding allowance allocations. The workpiece surface, which had undergone carburization and quenching treatment, exhibited microhardness beyond 600 HV, gradually decreasing with the increasing measurement depth. Up to a measurement depth of 1100 μm, the hardness value fluctuated around 450 HV. The curve shows that the peak microhardness value of each sample was higher than 550 HV, within a depth range of 100 μm to 700 μm. Comparing the microhardness curve of the workpiece surface before grinding (as shown in Figure 14a), the initial effective carburized layer thickness was approximately 1100 μm. Since the material removed by grinding was 400 μm, the change in the residual carburized layer thickness due to the grinding process was not significant.

The sample from trial P1 exhibited the highest peak microhardness on the ground subsurface, reaching close to 650 HV at a depth of 200 μm, which was 15 HV higher than the value at the same depth before grinding (at a depth of 600 μm). The increased hardness in trial P1 suggests the occurrence of work hardening during grinding. It is worth noting that in all the curves, the hardness value at a depth of 100 μm was lower than that at 200 μm. This may be because the measured indentation size was relatively large when located at the edge of the gear material and the resin, resulting in a smaller measured value than the actual one. For the other samples except the one from trial P1, another possible reason was the occurrence of thermal softening within a depth of 200 μm below the surface layer. The change in microhardness on the ground subsurface was the combined result of grinding heat effects and mechanical effects during grinding. When the thermal effect was dominant, the material’s hardness would be reduced, while the mechanical effect on hardness was opposite to the thermal effect. Since the grinding temperature in trial P1 was relatively low, work hardening occurred due to the mechanical effect contributed by the grinding wheel. In contrast, the surface hardness curve obtained in trial P3 showed thermal softening during grinding, with a hardness value at a depth of 200 μm approximately 40 HV lower than the hardness value at a depth of 600 μm before grinding, consistent with the measurement results of the grinding temperature. The hardness gradient curves of the subsurface obtained from the other trials also exhibited slight thermal softening. The thermal softening effect was most pronounced in trial P7, with a reduction of about 30 HV at a depth of 200 μm. The difference in the hardness gradient among different trials indicates that the allocation mode of the grinding allowance covertly influences the microhardness gradient of the ground subsurface, based on the combined effects of the mechanical and thermal factors. 

### 3.5. Grinding Strategy Validation

In order to verify the results of the previous experiment, three 20Cr2Ni4A gear ring blanks marked A, B, and C were randomly selected at the manufacturing site for machining. The original allowance distribution strategy was selected for grinding gear A, and the optimized distribution strategy was used for gear B and gear C. The specific process parameters were shown in Table 3. During the grinding of gear B and gear C, the number of strokes in the rough grinding stage was reduced by increasing the grinding depth, and the spindle speed of the grinding wheel was increased appropriately.

**Figure 15:**
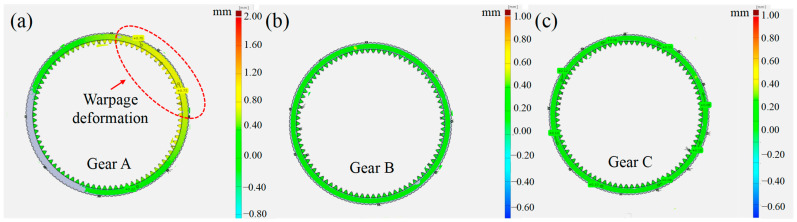
Deformation distribution of gears manufactured with different grinding strategies for (**a**) gear A, (**b**) gear B, and (**c**) gear C.

After grinding, the dimensions of the gears were recorded including cumulative tooth pitch deviation, tooth thickness deviation, profile deviation, and lead profile deviation. Improving the geometrical accuracy of gear dimensions can reduce transmission errors and heat production during operating, and it can also reduce mechanical shock and noise. The results are presented in Table 4 and Figure 15. The gear rings processed via the optimized strategy presented good dimension accuracy, verifying the feasibility of achieving higher dimensional accuracy while improving grinding efficiency.

## 4. Conclusions

(1) The allocation mode of the grinding allowance has a significant influence on the maximum grinding temperature. Multiple grinding passes with large cutting depths in the rough grinding and first semi-fine grinding stages result in a relatively higher temperature, and the corresponding grinding temperature model under different grinding allowance allocations was proposed.
(2) The allocation mode of the grinding allowance in the finish grinding stage affects the ground surface morphology, limiting the removal of material thickness (within 0.02 mm) in the fine grinding stage and making it difficult to polish the rough surface formed by rough grinding, with the surface roughness Ra exceeding 1.60 μm. Grain refinement and defects such as plastic deformation, dark layer, material pull-out, and adhesion, caused by thermal effects, are observed on the ground surface.
(3) Different grinding temperature levels result in variations in surface hardness. The trial with the lowest grinding temperature exhibited a work hardening effect, with an increase in microhardness of 15 HV in the ground subsurface. Conversely, the trial with the highest grinding temperature showed a thermal softening effect, with a decrease in microhardness of 40 HV within the subsurface layer.
(4) The feasibility of achieving higher dimensional accuracy while improving grinding efficiency was validated when using the optimized grinding strategy, with the distribution of allowance designed via gradual reduction according to the grinding pass/stroke sequence.

## Figures and Tables

**Table 1:** Chemical composition of 20Cr2Ni4A steel (wt.%).

C	Si	Mn	Cr	Ni	Fe	S
0.17–0.23	0.17–0.37	0.30–0.60	1.25–1.65	3.25–3.65	≥95.00	≤0.03

**Table 2:** Grinding depth and number of passes of different test plans.

Test Plans	Rough Grinding		Semi-Finishing Grinding 1		Semi-Finishing Grinding 2		Fine Grinding	
	a_p_ (mm)	Passes	a_p_ (mm)	Passes	a_p_ (mm)	Passes	a_p_ (mm)	Passes
P1	0.020	12	0.015	8	-	-	0.010	4
P2	0.025	8	0.020	6	0.015	4	0.010	2
P3	0.025	14	0.015	2	-	-	0.010	2
P4	0.030	12	-	-	-	-	0.010	4
P5	0.030	10	0.020	4	-	-	0.010	2
P6	0.030	6	0.025	6	0.015	4	0.005	2
P7	0.035	4	0.030	4	0.015	4	0.010	2
P8	0.035	8	0.025	4	-	-	0.010	2
P9	0.035	2	0.030	6	0.020	6	0.015	2

**Table 3:** Grinding parameters of gears in on-site machining.

Gear	Strategy	Grinding Depth (mm)	Strokes	Spindle Speed (r/min)	Total Strokes
A	Rough grinding	0.015	12	2700	17
	Semi-finish grinding	0.013	4	2400	
	Fine grinding	0.010	1	2100	
B	Rough grinding	0.025	2	2700	14
	Semi-finish grinding	0.020	6	2700	
	Semi-finish grinding	0.014	4	2400	
	Fine grinding	0.01	2	2100	
C	Rough grinding	0.018	8	2700	14
	Semi-finish grinding	0.015	4	2700	
	Fine grinding	0.01	2	2700	

**Table 4:** Results of dimensional accuracy of machined gears.

Gear	Cumulative Pitch Deviation (mm)	Tooth Thickness Deviation (mm)	Profile Deviation (mm)	Lead Profile Deviation (mm)
A	0.0297	0.0309	0.0083	0.0099
B	0.0137	0.0131	0.0069	0.0092
C	0.0186	0.0164	0.0056	0.0088

## Data Availability

The data that support the findings of this study are available from the corresponding author upon reasonable request.
